# Self Organization of Binary Colloidal Mixtures via Diffusiophoresis

**DOI:** 10.3389/fchem.2022.803906

**Published:** 2022-03-10

**Authors:** Lijie Lei, Shuo Wang, Xuemao Zhou, Salah Eddine Ghellab, Guanhua Lin, Yongxiang Gao

**Affiliations:** ^1^ Institute for Advanced Study, Shenzhen University, Shenzhen, China; ^2^ Institute of Microscale Optoelectronics, Shenzhen University, Shenzhen, China

**Keywords:** Brownian dynamics, diffusiophoresis, self-assembly, active colloids, nonreciprocal interaction

## Abstract

Catalytic activity of the colloids and chemotactic response to gradients of the chemicals in the solution leads to effective interaction between catalytic colloids. In this paper, we simulate mixtures of active and passive colloids via a Brownian dynamics algorithm. These particles interact via phoretic interactions, which are determined by two independent parameters, surface activity and surface mobility. We find rich dynamic structures by tuning passive colloids’ surface mobility, size, and area fractions, which include schools of active colloids with exclusion zone, yolk/shell cluster, and stable active–passive alloys to motile clusters. Dynamical cluster can also be formed due to the nonreciprocity of the phoretic interaction. Increasing the size ratio of passive colloids to active colloids favors the phase separation of active and passive colloids, resulting in yolk/shell structure. Increasing the area fraction of active colloids tends to transfer from dynamical clusters into stable alloys. The simulated binary active colloid systems exhibit intriguing nonequilibrium phenomena that mimic the dynamic organizations of active/passive systems.

## 1 Introduction

Active matter can harvest energy from the environment for mechanical motion, such as bacterial suspension (Ishikawa and Hota, 2006; Cates et al., 2010; McCarter, 2010; Marchetti et al., 2013; Singh et al., 2017), fish schools, and animal flocks (Ballerini et al., 2008; Moussaïd et al., 2009). These biological systems have inspired the design of artificial swimmers experimentally with different sources of energy, including chemical (Howse et al., 2007), electromagnetic (Creppy et al., 2016), acoustic (Ahmed et al., 2014), magnetic (Wang M. et al., 2013; Tierno, 2014; Wang et al., 2021), or thermal (Jiang et al., 2010) energy. Systems driven by chemicals have obvious advantages over others since they require no moving parts and can be free from external actuations (Varma et al., 2018). Chemically active colloids (Vicario et al., 2005; Niu et al., 2018; Yu et al., 2018; Shah et al., 2020; Zhang et al., 2021) can exhibit chemotaxis in response to gradients of chemicals that they themselves produce or consume. Concentration gradients of a solute along the surface of a chemotactic particle in a fluid produces pressure difference resulting in a slip flow parallel to the surface, so that the particle moves through the fluid in the direction opposite the slip velocity (Saha et al., 2019). This phenomenon is known as diffusiophoresis (Anderson, 1989).

Catalytic particles interact with each other through their influence and response on the chemical’s concentration field resulting in a wide variety of self-organization in heterogeneous populations of microorganisms (Keller and Surette, 2006), cell–cell communication (Friedl and Gilmour, 2009), aggregation of enzymes (Wu et al., 2015; Sun et al., 2017), and assembly of active materials from catalytical colloids (Palacci et al., 2013; Qin et al., 2017). Rich collective phenomena, such as flocking and schooling, have been observed in experiments and simulations (Mei et al., 2011; Lee et al., 2014; Choudhury et al., 2015; Agudo-Canalejo and Golestanian, 2019; Ouazan-Reboul et al., 2021). However, these studies have focused mostly on the mixture of active particles, while much less has been done for active/passive mixtures. Experimental results revealed that the active particles can collect the passive particles (Wang W. et al., 2013). Specifically, Ibele et al. (Ibele et al., 2009) have reported that photo-inactive silica particles surround active silver chloride (AgCl) particles while maintaining a small exclusion zone when the UV light is on. Meanwhile, some theoretical studies have predicted that a small fraction of active colloids can promote crystallization in passive hard-sphere glasses (Ni et al., 2014) or that a small fraction of active colloids can induce phase separation and self-assembly in passive colloids (Stenhammar et al., 2015), suggesting the crucial role of area fraction in controlling the collective behavior of the binary mixture. These studies also provide insights into the active–passive binary colloidal systems and demonstrate the importance of nonreciprocity in phase transitions (Fruchart et al., 2021). However, the role of surface properties and size of colloids on the dynamics and self-organization of the active–passive binary system remains unexplored.

In this work, we numerically investigated the collective behavior of binary active/passive colloids systems in which colloidal particles interact and respond via diffusiophoresis. Here the active colloids present the catalytic active colloids, such as Pt and hematite (Howse et al., 2007; Zhou et al., 2021), which will decompose the chemicals, for example, H_2_O_2_ in the solution, while the passive colloids will not decompose the chemicals, but they will respond to the chemical gradient, such as TPM particles (Zhou et al., 2021). By altering the surface parameters, size ratio, and area fractions of each component, we demonstrate that the binary mixtures can self-organize into structures, such as the yolk/shell, binary active/passive alloy, dynamical clusters, and moving clusters. Note that phoretic interactions between particles are not restricted to be reciprocal and are inherently non-equilibrium, which, therefore, may lead to binary colloidal systems with distinct structural diversities.

## 2 Methodology

Diffusiophoretic motion can be characterized by two distinct physicochemical properties of the colloids. The first one is the surface activity *α*, which refers to the colloid’s ability to generate (*α* > 0) or consume (*α* < 0) solute molecules through chemical reactions on their surface, and *α* = 0 corresponds to passive colloids. The second one is surface mobility, also termed as phoretic mobility *μ*, which describes the ability of colloids in generating an effective slip motion in response to chemical gradients near the surface (Soto and Golestanian, 2014). *μ* < 0 (*μ* > 0) corresponds to the colloid moving toward a high (low) chemical concentration. Experimentally, these two parameters can be manipulated by modifying the surface chemistry of the colloidal particles (Lyu et al., 2021; Zhou et al., 2021).

To introduce our model, we consider two colloidal particles, colloid (1) and colloid (2), randomly in a quasi two-dimensional domain, with a radius *R*_1_ and *R*_2_. The surface parameters are *α*_1_, *α*_2_ and *μ*_1_, *μ*_2_. The concentration field *c*(*r*) around colloid 1 in 3D can be obtained by solving the diffusion equation

$$\nabla^{2}c=0,$$ (1)

with a boundary condition

$$-D\frac{\partial c}{\partial r}{|}_{r=R_{1}}=\alpha_{1},$$ (2)

where *D* is the diffusion coefficient of the solute chemicals. The resulting concentration field decays as $\frac{1}{r}$ ,

$$c\left(r\right)=\frac{\alpha_{1}R_{1}^{2}}{Dr}.$$ (3)

The diffusiophoretic motion applied to colloid 2 in this concentration field can be calculated by:

$$v_{2}=-\mu_{2}\nabla c\left(r\right)∼-\frac{\alpha_{1}\mu_{2}R_{1}^{2}}{Dr_{12}^{2}}.$$ (4)

where **r**_12_ = **r**_1_ − **r**_2_ and *r*_12_ = |**r**_12_|. Similarly, the diffusiophoretic motion of colloid 1 induced by colloid 2 is $v_{1}=-\mu_{1}\nabla c\left(r\right)∼-\frac{\alpha_{2}\mu_{1}R_{2}^{2}}{Dr_{12}^{2}}$ . We should note that **v**_1_ ≠ **v**_2_, in general, because *α*_1_ *μ*_2_ ≠ *α*_2_ *μ*_1_ even when the two colloidal particles are of equal size. This implies a broken action–reaction symmetry for interspecies interactions. We can interpret chemotactically that when *α* > 0, the colloid’s surface will generate the chemical in the solution, and when *μ* < 0, the colloid will move toward a high chemical concentration. Then it is quite direct that *α*_1_ *μ*_2_ > 0 indicates a repulsive interaction applied to colloid 2 induced by the chemical gradient imposed by colloid 1, while when *α*_1_ *μ*_2_ < 0, colloid 2 will be attracted by colloid 1. If two colloids form a dimer, then the overall velocity can be approximated by $V∼\alpha_{1}\mu_{2}\left(\frac{R_{1}^{2}r_{12}}{D|r_{12}{|}^{3}}\right)-\alpha_{2}\mu_{1}\left(\frac{R_{1}^{2}r_{12}}{D|r_{12}{|}^{3}}\right)$ , and for equal-sized colloids, the whole pair moves with a velocity simply proportional to (*α*_1_ *μ*_2_ − *α*_2_ *μ*_1_). This non-equilibrium property is important for forming moving structures.

### 2.1 Equation of Motion

Based on the presented diffusiophoresis model, the equation of motion governing the movement of the colloids can be formulated as the following stochastic equation:

$$\frac{dr_{i}}{dt}=\underset{j\neq i}{\sum }\frac{\alpha_{j}\mu_{i}R_{j}^{2}}{6\pi D}\frac{r_{ji}}{|r_{ij}{|}^{3}}+\xi_{i}\left(t\right)=V_{0}\underset{j\neq i}{\sum }{\hat{\alpha }}_{j}{\hat{\mu }}_{i}\frac{\sigma_{j}^{2}r_{ji}}{|r_{ij}{|}^{3}}+\xi_{i}\left(t\right),$$ (5)

where **r**_*i*_ is the position of colloid *i*, *σ*_*j*_ is the diameter of colloid *j*, and $V_{0}=\frac{\alpha_{0}\mu_{0}}{24\pi D}$ is the overall velocity scale. Using *α*_0_ and *μ*_0_ as characteristic values for the surface activity and surface mobility, the dimensionless surface activity and surface mobility are defined as $\tilde{\alpha }=\alpha /\alpha_{0}$ and $\tilde{\mu }=\mu /\mu_{0}$ . The first term in Eq. 5 is the velocity induced by the phoretic interaction between colloid *i* and colloid *j* in the far-field approximation, and *ξ*_*i*_(*t*) is a random velocity applied to particle *i* at time *t*, a white noise with intensity of *D*_*c*_, which is proportional to *k*_*B*_ *T*, representing the effect of thermal motion. The dimensionless white noise intensity is further defined as ${\tilde{D}}_{c}=D_{c}/V_{0}\sigma$ . In general, colloidal particles in experiments cannot overlap. In our simulation, an electrostatic double-layer repulsion at a very short separation is applied to guarantee the hard-sphere boundary for the colloids. Periodic boundary conditions are used, and the interactions between colloids near crossing boundary are treated using the minimal image convention (Rapaport, 2004). As most of the experiments are confined to 2D, we confine the colloidal particles to a quasi-3D domain by limiting the center of the particle to move within half of the particle’s radius without considering real plates to avoid additional complexity. When the particles reach this imaginary boundary, they are bounced forward to the domain. This allows us to apply the formulas derived for three-dimensional systems to this quasi-3D condition. Note that we ignore the hydrodynamic interaction between particles and assume the concentration field will not be disturbed by the presence of other particles in our simulation (Soto and Golestanian, 2014).

## 3 Results and Discussion

In this study, we consider a binary mixture of active and passive colloids. The active colloids have nonzero surface activity ${\tilde{\alpha }}_{a}$ and mobility ${\tilde{\mu }}_{a}$, which means that they can generate a gradient in the solution and respond to the gradient in the solution. For passive colloids, they have zero surface activity ${\tilde{\alpha }}_{p}$, but they have a nonzero surface mobility ${\tilde{\mu }}_{p}$, and they will also respond to the gradient generated by the active colloids. We can see that the passive colloids will have no effect on the active colloids, while the active colloids will attract $\left({\tilde{\mu }}_{p}<0\right)$ /repel $\left({\tilde{\mu }}_{p}>0\right)$ the passive colloids, which means that the interactions between the active and passive colloids are nonreciprocal. The area fraction is defined as $\phi_{a}=\frac{N_{a}\pi R_{a}^{2}}{L^{2}}$ and $\phi_{p}=\frac{N_{p}\pi R_{p}^{2}}{L^{2}}$ for active and passive colloids, respectively, where *N*_*a*_, *R*_*a*_ and *N*_*p*_, *R*_*p*_ are the number and radius of active and passive colloids, respectively. The total area fraction of all the colloids is, therefore, *ϕ*_*tot*_ = *ϕ*_*a*_ + *ϕ*_*p*_. We fix the surface parameters of the active colloids taking ${\tilde{\alpha }}_{a}=1.0$ and ${\tilde{\mu }}_{a}=-1.0$ so that active colloids will release chemicals into the solution, and their intraspecies interaction is attractive only, since ${\tilde{\alpha }}_{a}{\tilde{\mu }}_{a}<0$ . We vary the surface mobility of the passive colloids so that the interaction between active and passive colloids can be tuned from strong repulsion to strong attraction (compared with the strength of intraspecies attraction of active colloids) progressively, as summarized in Table 1.

**TABLE 1:** Phoretic interaction applied to passive colloids by active colloids with different surface parameters (S. R., strong repulsion; W. R., weak repulsion; W. A., weak attraction; E. A., equal attraction; S. A., strong attraction.).

Active colloid	Passive colloid				
	${\tilde{\mu }}_{p}=1.0$	${\tilde{\mu }}_{p}=0.1$	${\tilde{\mu }}_{p}=-0.2$	${\tilde{\mu }}_{p}=-1.0$	${\tilde{\mu }}_{p}=-2.0$
${\tilde{\alpha }}_{a}=1.0,{\tilde{\mu }}_{a}=-1.0$	S. R	W. R	W. A	E. A	S. A

In this section, we first summarize the typical collective behavior of the binary mixture in Section 3.1. In Section 3.2, we present the results of active/passive mixtures of equal size and equal area fraction with varying surface mobilities of the passive colloids. In Section 3.3, we study the effect of relative area fraction with fixed total area fraction of the colloids. In Section 3.4, we alter the size ratio $\beta =\frac{R_{p}}{R_{a}}$ and study its effect on the collective behavior of the binary mixture.

### 3.1 Typical Collective Behavior

**FIGURE 1:**
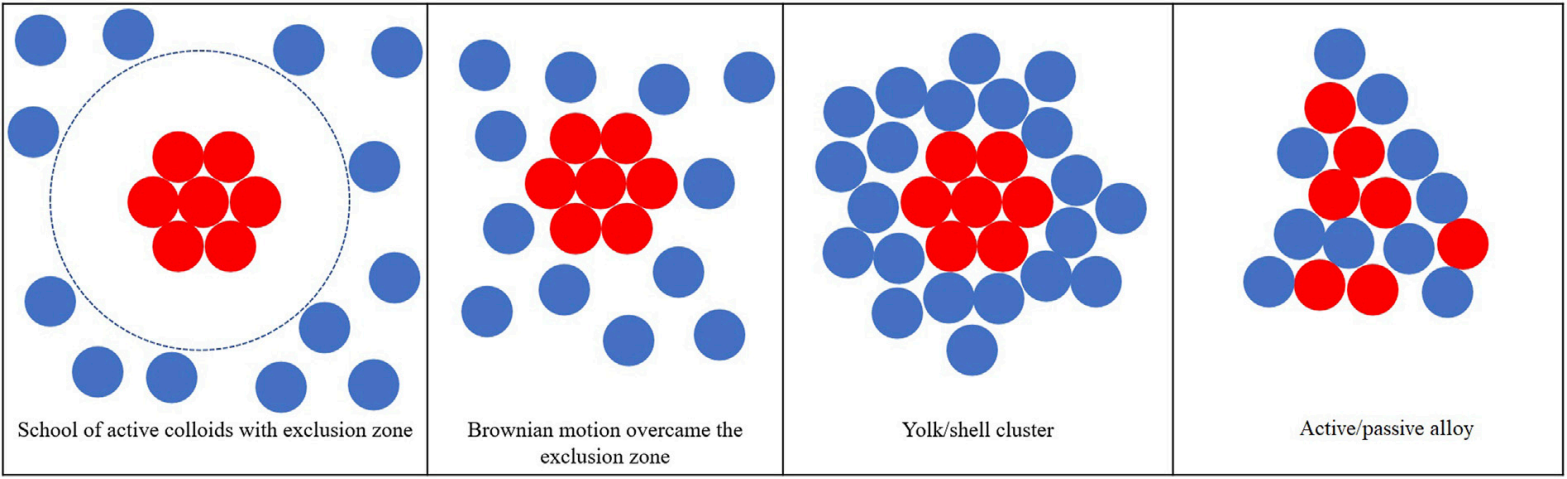
Typical collective behavior of active/passive mixture with different surface mobilities of the passive colloids.

The general structure formed by the binary colloidal system is plotted in Figure 1, which can be categorized into four groups: 1) school of active colloids (active colloids aggregate together resulting in a region with higher active colloids concentration) with an exclusion zone that passive colloids cannot enter because of the strong repulsion applied to the passive colloids; 2) active colloids cluster surrounded by the passive colloids, in this scenario the phoretic force applied to passive colloids is weak repulsion which can be overcome by the Brownian motion; 3) yolk/shell structure with active cluster core and passive colloids shell are attached to the core due to the weak attraction applied to the passive colloids; 4) active–passive alloys formed when the attraction applied to the passive colloids are of the same magnitude compared to the active–active phoretic attraction.

### 3.2 Effect of Varying Surface Mobility of Passive Colloids

**FIGURE 2:**
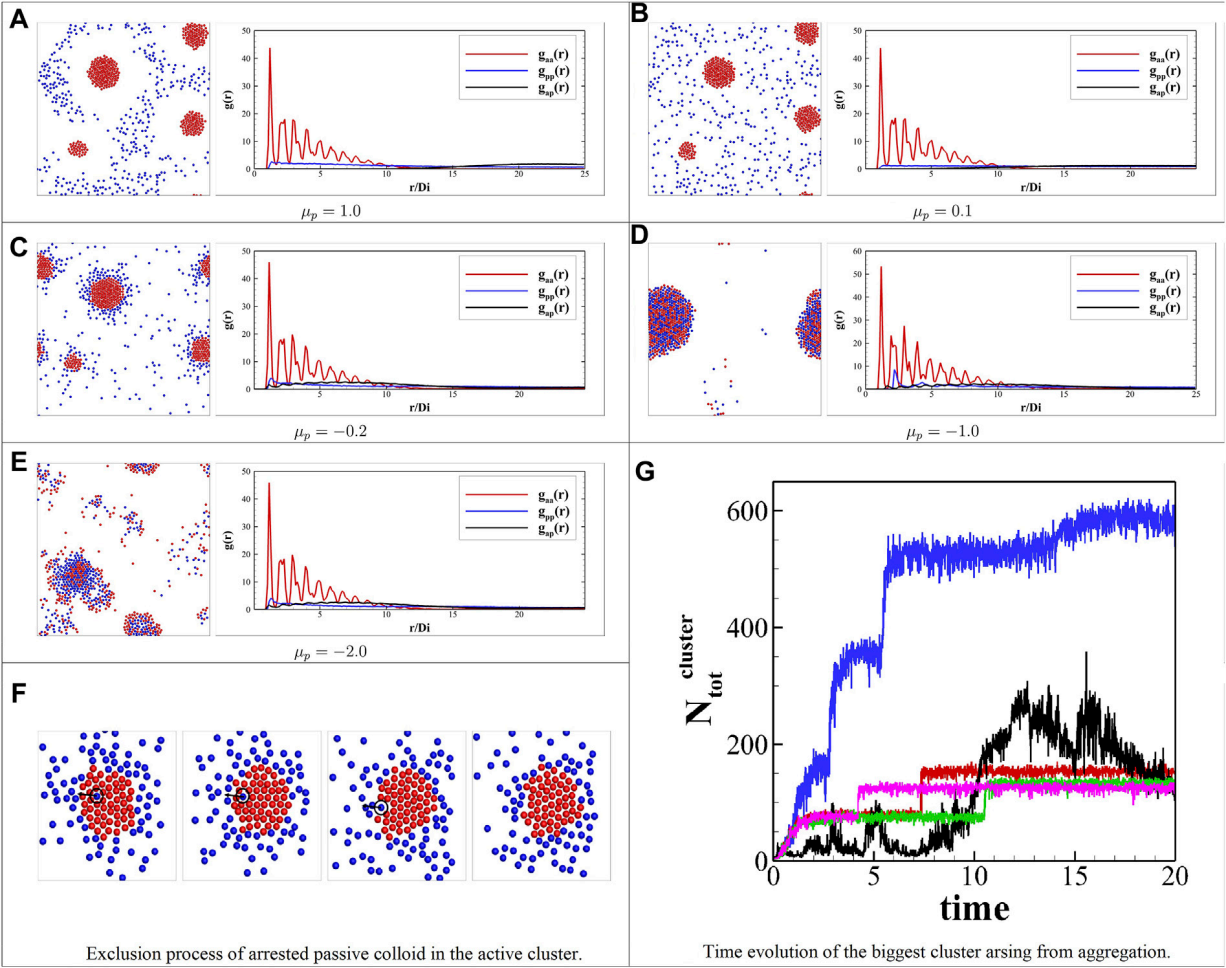
Self-organization observed in the simulation with different surface parameters of passive colloids’ mobility. The left side shows the snapshots of **(A)** a school of active colloids with an exclusion zone, **(B)** active colloid clusters surrounded by the passive colloids, **(C)** yolk/shell structure, **(D)** binary active/passive alloy, **(E)** dynamical clusters. The right side plots the corresponding pair correlation functions from a–e; the red line *g*_*aa*_(*r*) is the pair correlation function for active–active colloids; similarly, the blue line *g*_*pp*_(*r*) is for passive–passive colloids, and the black line *g*_*ap*_(*r*) is for active–passive colloids. The distance is scaled by the diameter (2*R*_*a*_) of the active colloid **(F)** exclusion of a single trapped passive colloid in the active cluster. **(G)** Time evolution of stoichiometry of the biggest cluster arising from aggregation for different cases; ${\tilde{\mu }}_{p}=1.0$ is the green line; ${\tilde{\mu }}_{p}=0.1$ is the purple line; ${\tilde{\mu }}_{p}=-0.2$ is the red line; ${\tilde{\mu }}_{p}=-1.0$ is the blue line; ${\tilde{\mu }}_{p}=-2.0$ is the black line.

To investigate the effect of the surface mobility of passive colloids, we consider the case of *β* = *R*_*p*_/*R*_*a*_ = 1 and *ϕ*_*a*_ = *ϕ*_*p*_ = 5*%*. Figure 2 shows the snapshots of the binary colloid systems’ collective behavior at different passive colloids’ surface mobility ${\tilde{\mu }}_{p}$ and their corresponding pair correlation functions. Since ${\tilde{\alpha }}_{a}{\tilde{\mu }}_{a}=-1.0$ , active colloids will attract each other resulting in the aggregation of active colloids for all the cases. We can see from the pair correlation functions of active colloids in Figures 2A–E that periodic peaks in *g*_*aa*_(*r*) indicate the crystal-like arrangement of active colloids. The active colloids always form active clusters, which tend to be crystal-like. Varying the mobility of passive colloids from ${\tilde{\mu }}_{p}=1.0$ to ${\tilde{\mu }}_{p}=-2.0$ corresponds to the interaction induced by the active colloids from strong repulsion to strong attraction (passive colloids chemotactically move toward a higher chemical concentration and shift to move toward a lower chemical concentration). When the phoretic force applied to the passive colloids is a strong repulsion ${\tilde{\mu }}_{p}=1.0$ , as in Figure 2A, the passive colloids cannot approach the active colloid cluster resulting an exclusion zone between the active cluster and the passive colloids. This phenomenon is in good agreement with the previous experiment results (Ibele et al., 2009) due to diffusiophoresis. If the repulsion is small ${\tilde{\mu }}_{p}=0.1$ , as in Figure 2B, generally, the passive colloids are repelled by the active cluster, but the passive colloids may approach the active cluster since the Brownian motion can overcome the small repulsion. The whole system behaves like a crystal core formed by the active colloids and gas phase by the randomly moving passive colloids. When the surface mobility of passive colloids is further decreased, the phoretic force applied on the passive colloids by the active colloids will alter from repulsion to attraction once ${\tilde{\mu }}_{p}<0$ . In this region, cases become more complicated for binary active colloid systems. First, when the attraction on passive colloids is weak, e.g., ${\tilde{\mu }}_{p}=-0.2$, as in Figure 2C, the passive colloids do show the attraction applied by the active colloids. However, the active colloids and passive colloids still separate into different phases since the passive colloids feel an attraction from the active colloids, but the difference between the attraction strength is still strong enough to separate the two components into two phases resulting in the yolk/shell structure. Then the attraction applied by the active colloids to the passive colloids is further increased until ${\tilde{\mu }}_{p}=-1.0$ , which means that the attraction between active colloids is the same as the attraction applied to the passive colloids; the active colloids and passive colloids will collapse together. The pair correlation functions between active–active, active–passive, and passive–passive colloids almost collapsed as shown in Figure 2D, which indicates that in this situation, active colloids and passive colloids behave almost identically in the active/passive alloy due to the equal magnitude attraction despite the passive colloids having no effective interaction applied to the active colloids. Further increase in the attraction is applied to the passive colloids by the active colloids, i.e., ${\tilde{\mu }}_{p}=-2.0$ in Figure 2E. The passive colloids still approach the active colloids. Since the passive colloids apply no attraction to the active colloids, this nonreciprocity will lead to the movement of locally attached active and passive colloids (Agudo-Canalejo and Golestanian, 2019; Lei et al., 2020), forming this dynamical cluster in which the active and passive colloids aggregate to form clusters, and these formed clusters will break due to the nonreciprocity (see Support Supplementary Video S1). In addition, at the initial stage, part of the passive colloids may be arrested by the active cluster because of the random distribution of the passive colloids, but the interaction difference can exclude the passive colloids from the near-boundary region to outside of the active cluster as plotted in Figure 2F, which are observed for ${\tilde{\mu }}_{p}=0.1,-0.2$ . Figure 2 plots the time evolution of stoichiometry of the biggest cluster arising from aggregation (Colloids within 0.1*R*_*a*_ are considered to belong to one cluster). As can be seen, at the initial stage, the total number of colloids in the biggest cluster starts to increase for all the cases since the active colloids attract each other. When two nearby clusters merged into one cluster, the number of colloids in the biggest cluster will increase immediately, which is observed for ${\tilde{\mu }}_{p}=1.0,0.1,-0.2$ and −1.0. Moreover, since for ${\tilde{\mu }}_{p}=1.0$ and 0.1, the active colloids will repulse the passive colloids, and only active colloids aggregate to form the cluster. Thus, the total numbers of the colloids in the biggest clusters are almost the same for these two cases. For ${\tilde{\mu }}_{p}=-0.2$, the yolk/shell structure formed at the final stage. Thus, the total number of colloids in the biggest cluster is bigger than the ${\tilde{\mu }}_{p}=1.0,0.1$ cases. By further decreasing the mobility of the passive colloids to ${\tilde{\mu }}_{p}=-1.0$ , most of the colloids aggregate to the big cluster; thus, the total number of the biggest cluster will grow to almost 600, while for ${\tilde{\mu }}_{p}=-2.0$ , the number of colloids in the biggest cluster varies with time since the forming and breaking of the dynamical cluster. The current results suggest that it is possible to control the structure formed by catalytic active and passive colloids by changing the surface mobility of the passive colloids to realize the specific structure, such as the yolk/shell structure, which is promising for nanoreactors, drug delivery, and lithium-ion batteries (Camargo et al., 2007; Liu et al., 2011; Watanabe et al., 2017).

### 3.3 Effects of Relative Area Fraction

**FIGURE 3:**
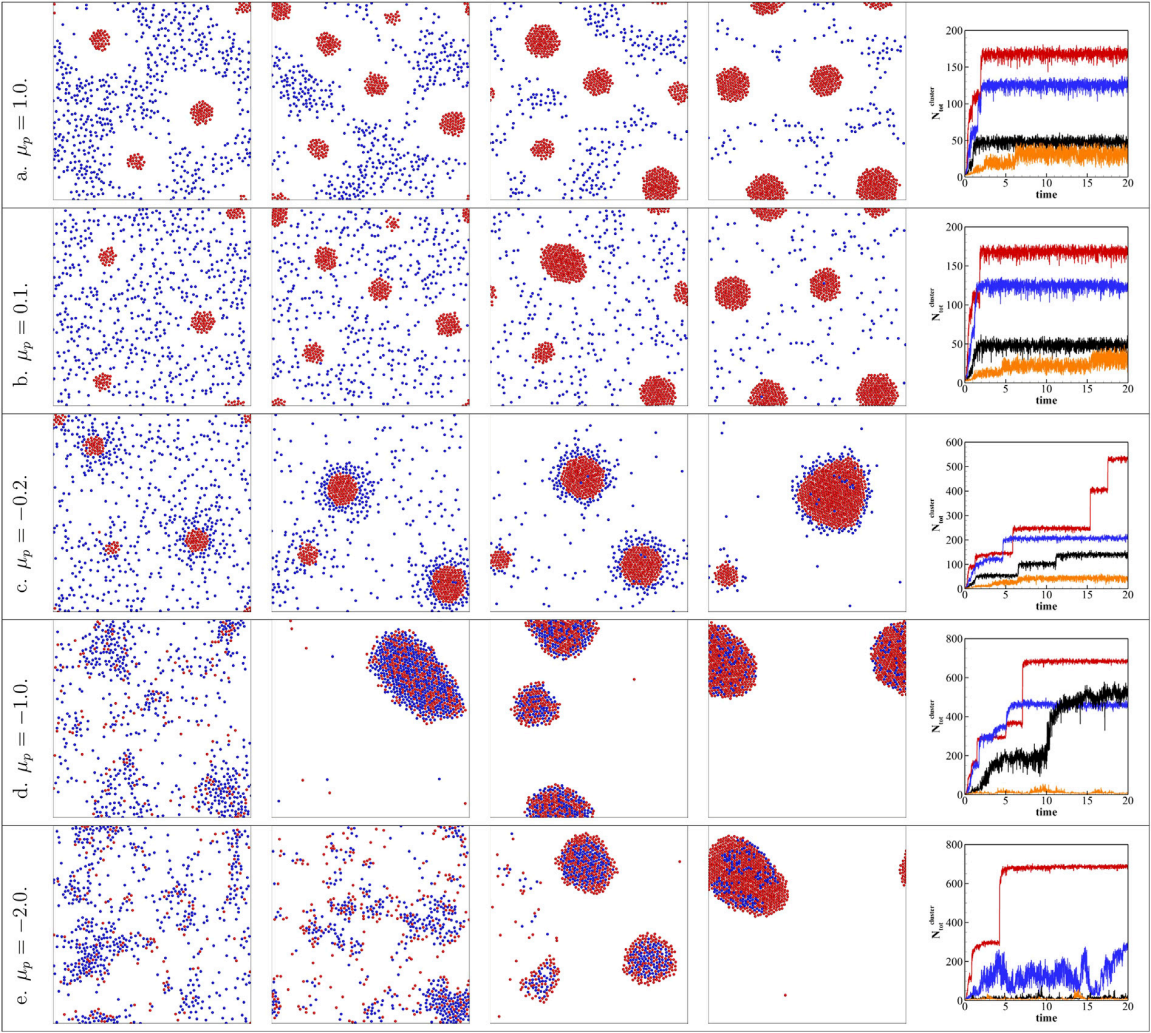
Keeping the total area fraction *ϕ*_*tot*_ = 10*%*, increasing *ϕ*_*a*_ = 2*%* to *ϕ*_*a*_ = 8*%* with different passive colloid surface mobilities ${\tilde{\mu }}_{p}$ varies from 1.0 to − 2.0. **(A)** ${\tilde{\mu }}_{p}=1.0$ , **(B)** ${\tilde{\mu }}_{p}=0.1$ , **(C)** ${\tilde{\mu }}_{p}=-0.2$ , **(D)** ${\tilde{\mu }}_{p}=-1.0$ , **(E)** ${\tilde{\mu }}_{p}=-2.0$ . The area fractions of each species of colloids are specifically *ϕ*_*a*_ = 2*%*, *ϕ*_*p*_ = 8*%*; *ϕ*_*a*_ = 4*%*, *ϕ*_*p*_ = 6*%*; *ϕ*_*a*_ = 6*%*, *ϕ*_*p*_ = 4*%*; and *ϕ*_*a*_ = 8*%*, *ϕ*_*p*_ = 2*%* from left to right. The fifth column shows the time evolution of the stoichiometry of the biggest cluster arising from aggregation mixtures. *ϕ*_*p*_ = 2*%* is red, *ϕ*_*p*_ = 4*%* is blue, *ϕ*_*p*_ = 6*%* is black, and *ϕ*_*p*_ = 8*%* is orange.

Varying the area fraction of the active colloids *ϕ*_*a*_ while fixing the total area fraction *ϕ*_*tot*_ can also affect the collective behavior of the active/passive binary mixture. In this work, we keep the total area fraction *ϕ*_*tot*_ = 10*%* as a constant and increased the area fraction of active colloids from *ϕ*_*a*_ = 2*%* to *ϕ*_*a*_ = 8*%*. Figure 3 plots the results for different *ϕ*_*a*_ and ${\tilde{\mu }}_{p}$ . For ${\tilde{\mu }}_{p}>0$ , passive colloids are repelled by the active colloids leading to the formation of active clusters with (Figure 3A) or without (Figure 3B) an exclusion zone depending on the strength of the repulsion. Increasing the area fraction of active colloids *ϕ*_*a*_ results in increasing the size of active clusters without changing the phase behavior, as shown in Figures 3A, B. At the initial stage, the cluster size will increase. In these two cases, only the active colloids will aggregate together to form the cluster. Thus, increasing the area fraction of active colloids will increase the total number of colloids in the biggest cluster, and cluster merge happened only at *ϕ*_*a*_ = 8*%*. For low *ϕ*_*a*_ cases, the formed active clusters are too far away from each other and will not merge.

However, for ${\tilde{\mu }}_{p}<0$, altering the area fraction of active colloids affects the phase behavior. For ${\tilde{\mu }}_{p}=-0.2$ in Figure 3C, the yolk/shell structure has a larger active core at high *ϕ*_*a*_. This is because the active core will merge at higher *ϕ*_*a*_. As can be seen at *ϕ*_*a*_ = 2*%*, the total number of colloids in the biggest cluster increases smoothly to a stable number, while for *ϕ*_*a*_ = 4, 6, 8*%*, the total number of the biggest cluster all observed a sudden increase, which indicates the merge of clusters. Also, there are more passive colloids trapped and arrested in the active core as *ϕ*_*a*_ increases. For ${\tilde{\mu }}_{p}=-1.0$ in Figure 3D, when *ϕ*_*a*_ = 2*%*, the colloids exhibit lose aggregation and form dynamical clusters similar to those in Support Supplementary Video S1. Since there is no stable cluster formed, the number of colloids in the biggest cluster is very small. By increasing *ϕ*_*a*_ above 4*%*, the dynamical cluster transformed into motile clusters of mixed colloids, which will eventually merge into a large motile cluster collecting all the colloids (see Support Supplementary Video S2). The number of colloids in the biggest cluster increases at the initial stage and merge of clusters is observed. For *ϕ*_*a*_ = 4, 6*%*, at the final stage, there are still some scattered colloids or small clusters, while for *ϕ*_*a*_ = 8*%*, almost all the colloids are in the big cluster, thus, reaching a highest total number of colloids in the biggest cluster. A similar observation has been reported in previous experimental studies (Buttinoni et al., 2013; Mognetti et al., 2013). For ${\tilde{\mu }}_{p}=-2.0$, in Figure 3E, the transition between dynamical clusters to the motile cluster has also been observed, but the transition point shifts to about *ϕ*_*a*_ = 6*%*. This result provides us a method to control the structure of binary mixtures by controlling the fraction of each component to realize either the dynamical or motile cluster.

### 3.4 Effects of Size Ratio

**FIGURE 4:**
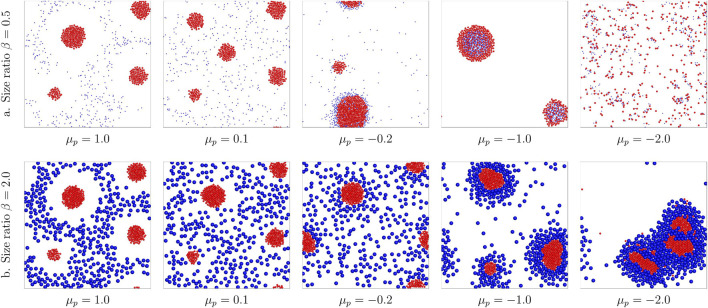
Comparison of binary structures formed by different size ratios *β* = 0.5, 2.0 with varying passive colloid mobilities ${\tilde{\mu }}_{p}$ from 1.0 to − 2.0. **(A)** *β* = 0.5; the binary structure varies from a school of active colloids with exclusion zone at ${\tilde{\mu }}_{p}=1.0$ , active cluster core with passive gas phase at ${\tilde{\mu }}_{p}=0.2$ , yolk/shell structure with trapped passive colloids at ${\tilde{\mu }}_{p}=-0.2$ , passive colloids enter the active clusters at ${\tilde{\mu }}_{p}=-1.0$ and dynamical active molecules at ${\tilde{\mu }}_{p}=-2.0$. **(B)** *β* = 2.0; the binary structure varies from a school of active colloids with exclusion zone at ${\tilde{\mu }}_{p}=1.0$ , active cluster core with passive gas phase at ${\tilde{\mu }}_{p}=0.2$ , yolk/shell structure with trapped passive colloids at ${\tilde{\mu }}_{p}=-0.2,-1.0$ and − 2.0.

Phoretic interactions also affected by the size of the colloids (Piazza and Parola, 2008; Ouazan-Reboul et al., 2021). Hence, we further investigate the effect of size ratio $\beta =\frac{R_{p}}{R_{a}}$ . Here, we consider two cases with *β* = 0.5, 2.0, corresponding to the active colloids are two times larger or smaller than the passive ones, respectively. Figures 4A,B plot the results of different size ratios with different *μ*_*p*_. It is shown that when the phoretic force applied on the passive colloids is repulsive, i.e., ${\tilde{\mu }}_{p}>0$ , the binary mixture phase separates into active clusters coexisting with the colloidal gas of passive colloids with $\left({\tilde{\mu }}_{p}=1.0\right)$ or without $\left({\tilde{\mu }}_{p}=0.1\right)$ an exclusion zone, similar to the behavior of the equal-sized system. However, when the phoretic force applied on the passive colloids are attractive, the size ratio shows a noticeable effect on the structure of the binary system. First, for ${\tilde{\mu }}_{p}=-0.2$ , when *β* = 0.5, there are more passive colloids arrested in the active cluster, while when *β* = 2.0, the passive colloids will not be arrested in the active clusters. This is because for *β* = 0.5, the decreased size of passive colloids leads to a reduced contact distance between active and passive colloids, i.e., a reduced *r*_12_ = *r*_*ap*_ = *R*_*a*_ + *R*_*p*_ = 1.5*R*_*a*_ in Eq. 5; however, for *β* = 2, increased *R*_*p*_ results in a larger *r*_*ap*_ = *R*_*a*_ + *R*_*p*_ = 3*R*_*a*_. Considering that the interspecies attraction is proportional to ${\tilde{\alpha }}_{a}{\tilde{\mu }}_{p}/|r_{ap}{|}^{2}$ , *β* = 0.5 results in a stronger interspecies attraction, while *β* = 2 leads to a reduced attraction strength. The intraspecies attraction between active colloids $∼{\tilde{\alpha }}_{a}{\tilde{\mu }}_{a}/|r_{aa}{|}^{2}=-1.0{\tilde{\alpha }}_{a}/\left(2R_{a}\right){}^{2}$ remains constant; thus, for larger *β*, the active and passive colloids are easier to separate since the difference between the intraspecies attraction of active colloids and the interspecies attraction increased, while smaller *β* reduces such difference so that the passive colloids are more likely to be arrested/mixed in the active cluster. Based on this analysis, it can be understood that for ${\tilde{\mu }}_{p}=-1.0$ , when *β* = 0.5, the passive colloids are fully arrested inside the active clusters, while well separated yolk/shell structures can be found at *β* = 2.0. These two cases are totally different from the equal-sized case as plotted in Figure 2D, which forms a binary alloy. By further decreasing the mobility of passive colloids to ${\tilde{\mu }}_{p}=-2.0$ , at *β* = 2.0, we still observe well-separated yolk/shell structures, while for *β* = 0.5, dynamically active molecules can be found (see Support Supplementary Video S3). These active colloidal molecules keep forming and breaking resulting in an unsteady system, which is similar to previously reported behavior induced by the nonreciprocal interaction (Soto and Golestanian, 2014; Lei et al., 2020).

## 4 Concluding Remarks

We have presented a systematic investigation for mixtures of chemically active and passive colloids via a diffusiophoresis model, which not only captures collective behavior observed experimentally but also reveals some intriguing new self-organizations. We demonstrate that the binary active/passive colloids systems can self-organize into schools of active colloids with exclusion zone, active crystal core surrounded by passive colloids, yolk/shell structure, dynamical clusters, and motile clusters. Exclusion zone is formed when the passive colloids feel strong repulsion from the active colloids, which are in accordance with previous experiment results (Ibele et al., 2009). In addition, active colloids and passive colloids will separate into two phases when the attraction difference is large enough resulting in the yolk/shell structure. The binary mixture may collapse to a stable mixture when the attractive interaction strength is of comparable magnitude. Further increasing the phoretic attraction applied to the passive colloids will lead to the formation of dynamical clusters. We further investigate the effects of size ratios and specific fractions of the components. Increasing the size of passive colloids favors the formation of yolk/shell structures for the binary colloid system. We also showed that at low active colloid fraction, dynamical clusters may form due to the nonreciprocal interaction between these colloids, and increasing the fraction of active colloids results in the formation of motile clusters. These clusters can even further merge into a single big cluster collecting all the colloids. These predicted structures should be applicable to a wide variety of active–passive systems, for example, yolk/shell structures are reported to be useful in delivery and lithium-ion batteries for their tailorability and functionality in both the cores and hollow shells (Liu et al., 2011).

## Data Availability

The raw data supporting the conclusion of this article will be made available by the authors, without undue reservation.
