# Corrigendum: Self Organization of Binary Colloidal Mixtures via Diffusiohporesis

**DOI:** 10.3389/fchem.2022.898469

**Published:** 2022-04-21

**Authors:** Lijie Lei, Shuo Wang, Xuemao Zhou, Salah Eddine Ghellab, Guanhua Lin, Yongxiang Gao

**Affiliations:** ^1^ Institute for Advanced Study, Shenzhen University, Shenzhen, China; ^2^ Institute of Microscale Optoelectronics, Shenzhen University, Shenzhen, China

**Keywords:** Brownian dynamics, diffusiophoresis, self-assembly, active colloids, non-reciprocal interaction

In the original article, there were errors. There was a typo in the last word of the title. The corrected title is as follows:

“Self-Organization of Binary Colloidal Mixtures via Diffusiophoresis”

There was a typo in section **2 Methodology**, page 2, second paragraph, in which the plural form of “colloids” was used mistakenly. The corrected sentence is as follows:

“To introduce our model, we consider two colloidal particles, colloid (1) and colloid (2), randomly in a quasi two-dimensional domain, with a radius *R*_1_ and *R*_2_.”

There was a typo in section **2 Methodology**, page 2, third paragraph, in which the singular form of “particle” was used mistakenly. The corrected sentence is as follows:

“We should note that $v_{1}\neq v_{2}$ , in general, because $\alpha_{1}\mu_{2}\neq \alpha_{2}\mu_{1}$ even when the two colloidal particles are of equal size.”

There was a typo in section **2 Methodology**, “*2.1 Equation of Motion*”, page 3, in which a question mark appeared erroneously in a citation. The corrected citation is as follows:

“(Rapaport, 2004)”.

There was a typo in section **3 Results and Discussion**, “*3.1 Typical Collective Behavior*”, page 3, in which a space was missing after the last numbered point. The corrected point is as follows:

“4) active–passive alloys formed when the attraction applied to the passive colloids are of the same magnitude compared with the active–active phoretic attraction.”

There was a typo in section **3 Results and Discussion**, “*3.4 Effects of Size Ratio*”, page 6, in which “are” was used instead of “is”. The correct phrasing is as follows:

“when the phoretic force applied on the passive colloids is repulsive”.

The authors apologize for this error and state that this does not change the scientific conclusions of the article in any way. The original article has been updated.

